# New Endiandric Acid from *Beilschmiedia lumutensis* and Their Molecular Docking Study as α-amylase and α-glucosidase Inhibitors

**DOI:** 10.21315/tlsr2026.37.1.15

**Published:** 2026-03-31

**Authors:** Nur Amirah Saad, Muhammad Solehin Abd Ghani, Mohammad Tasyriq Che Omar, Mohd Azlan Nafiah, Unang Supratman, Desi Harneti, Cécile Apel, Marc Litaudon, Azeana Zahari, Khalijah Awang, Mohamad Nurul Azmi

**Affiliations:** 1 Natural Products and Synthesis Organic Research Laboratory (NPSO), School of Chemical Sciences, Universiti Sains Malaysia, 11800 USM Pulau Pinang, Malaysia; 2 Biological Section, School of Distance Education, Universiti Sains Malaysia, 11800 USM Pulau Pinang, Malaysia; 3 Department of Chemistry, Faculty of Science and Mathematics, Sultan Azlan Shah Campus, Universiti Pendidikan Sultan Idris, Proton City 35950 Tanjong Malim, Perak, Malaysia; 4 Department of Chemistry, Faculty of Mathematics and Natural Sciences, Universitas Padjadjaran, 45363 Jatinangor, Indonesia; 5 Institut de Chimie des Substances Naturelles, CNRS, UPR 2301, Université Paris-Saclay, 91198 Gif-sur-Yvette, France; 6 Department of Chemistry, Faculty of Science, Universiti Malaya, 50603 Kuala Lumpur, Malaysia

**Keywords:** Lauraceae, *Beilschmiedia lumutensis*, Cyclic Polyketides, Endiandric Acids, Molecular Docking, Lauraceae, Beilschmiedia lumutensis, Poliketida Siklik, Asid Endiandrik, Pengedokan Molekul

## Abstract

A preliminary study showed that an ethyl acetate extract of the bark from *Beilschmiedia lumutensis* (Lauraceae family) exhibited promising inhibition activities against α-amylase and α-glucosidase by *in vitro* assays. Subsequently, this extract revealed three new cyclic polyketides endiandric acids, namely lumutensic acid A-C (**1–3**). Their structures were elucidated by 1D and 2D NMR, FT-IR, HRESIMS spectroscopic data analysis and by comparison with literature data. The molecular docking study showed that lumutensic acid C (**3**) showed the highest binding affinity and stability with both enzymes, with strong hydrogen bonding and hydrophobic interactions, outperfoming the standard drug acarbose. These findings suggest that compound **3** could be a promising candidate for anti-hyperglycemic therapeutic development, providing further insight into the potential of *B. lumutensis* as a source of bioactive compounds.

## Abstrak

Kajian awal menunjukkan bahawa ekstrak etil asetat kulit batang Beilschmiedia lumutensis (famili Lauraceae) mempamerkan aktiviti perencatan yang memberangsangkan terhadap α-amilase dan α-glukosidase melalui asai in vitro. Seterusnya, ekstrak ini didapati mengandungi tiga asid endiandrik poliketida siklik baharu, iaitu asid lumutensik A-C (**1–3**). Struktur sebatian-sebatian ini telah dijelaskan melalui analisis data spektroskopi NMR 1D dan 2D, FT-IR, HRESIMS serta perbandingan dengan data literatur. Kajian pengedokan molekul menunjukkan bahawa asid lumutensik C (**3**) mempamerkan afiniti pengikatan dan kestabilan tertinggi terhadap kedua-dua enzim melalui ikatan hidrogen dan interaksi hidrofobik yang kuat, mengatasi ubat standard akarbosa. Dapatan ini mencadangkan bahawa sebatian **3** berpotensi menjadi calon yang baik untuk pembangunan terapeutik anti-hiperglisemik, sekaligus memberikan pemahaman lanjut mengenai potensi B. lumutensis sebagai sumber sebatian bioaktif.

**HIGHLIGHTS**

- The isolation and purification of the bark of *Beilschmiedia lumutensis* isolate 3 new endiandric acids.
- The ethyl acetate crude of *Beilschmiedia lumutensis* showed the potential to inhibit α-amylase and α-glucosidase with IC^50^ values of 7.90 ± 1.00 μg/mL and 21.91 ± 3.93 μg/mL, respectively.
- Lumutensic acid C (3) showed the highest binding affinity and stability with both enzymes, with strong hydrogen bonding and hydrophobic interactions, compared to acarbose (control).
- This is the first report on the chemical constituents of *Beilschmiedia lumutensis*.

## INTRODUCTION

*Beilschmiedia* is one of the largest pantropical genera in Lauraceae family which about 287 species being recognised mainly in Southeast Asia and Africa (Nishida 1999). In Southeast Asia, *Beilschmiedia* can be found in Vietnam, Myanmar, Thailand, Cambodia, Indonesia, Philippines, Malaysia and various island such as Sumatra and Java (Burkill 1966). This genus is rich in source of pharmacologically active chemical constituents, so they have been widely used in medicinal field (Iwu 1993). *Beilschmiedia lumutensis* (*B. lumutensis*) are trees 3 m to 15 m tall endemic to Peninsular Malaysia and Cambodia and vegetate in lowland and hill forests at 150 m to 200 m altitude and sometimes on sandstone or near streams.

**FIGURE 1:**
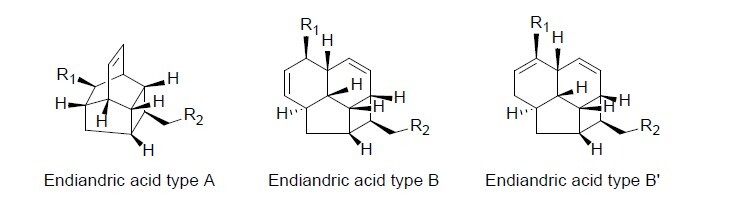
Endiandric acids main skeleton.

Endiandric acids, which possess a distinctive tetracyclic carbon skeleton formed with 11 or 13 carbon atoms including one or two double bonds and seven or eight sp^3^ hybridised methines, are exclusively produced by the *Beilschmiedia* and *Endiandra* species. These cyclic polyketides of types A, B or B′ (Fig. 1) have eight chiral centres and are generally isolated as a racemic mixture [αD] = 0° (Saad *et al*. 2022a). This is a rather unusual observation for naturally occurring compounds resulting from both shikimate and acetate pathways (Lenta *et al*. 2015; Saad *et al*. 2022a).

Previous studies show that 14 species of the genus *Beilschmiedia* have been investigated, in which 55 of the isolated compounds have been identify as endiandric acids. The genus Beilschmiedia still holds considerable potential for exploration due to the unique architecture of its compounds (Lenta *et al*. 2015; Saad *et al*. 2022a). In addition, previous studies showed that an ethyl acetate (EtOAc) extract of *B. lumutensis* bark exhibited inhibition activities against α-amylase and α-glucosidase with IC_50_ values of 7.90 ± 1.00 μg/mL and 21.91 ± 3.93 μg/mL, respectively (Tay *et al*. 2020). Based on this result and our previous study on various *Beilschmiedia* and *Endiandra* spp (Abu Bakar *et al*. 2020; Apel *et al*. 2014; Azmi *et al*. 2014; 2016; 2021; Saad *et al*. 2022b; Tay *et al*. 2020), we decided to further explore the potential of the chemical constituents isolated from *B. lumutensis* to inhibit the two mentioned enzymes. In addition, molecular docking study was carried out to investigate the binding interactions of the isolated compounds with the active residues of α-amylase and α-glucosidase. This is the first report on the chemical constituents of this plant.

## MATERIALS AND METHOD

### General

The commercial chemicals and reagents used in the isolation and characterisation process of all the isolated compounds are as follows: hexane, AR grade (QRëC); ethyl acetate, AR grade (QRëC); methanol, AR grade (QRëC); dichloromethane, AR grade (QRëC); acetone, AR grade (QRëC); ethanol, AR grade (QRëC); formic acid (Merck), sulphuric acid (Merck); acetonitrile, HPLC grade (Merck); methanol, HPLC grade (Merck); chloroform-d_1_ for NMR (Merck); methanol-d_4_ for NMR (Merck); silica gel 60 for column chromatography, 0.040 mm–0.063 mm and 0.063 mm–0.200 mm (Merck); TLC silica gel 60 F_254_, aluminium sheets, 20 cm × 20 cm (Merck). All solvents were used without further purification, unless otherwise stated.

Flash chromatographic techniques under controlled pressure were used with silica gel 60 (0.06 mm–0.2 mm) and subsequent purification with 230–400 and 70–230 silica gel mesh as well as pre-coated TLC silica 60 gel F_254_ 20 cm × 20 cm on aluminium sheet (Merck). The TLC sheet were observed under 254 nm UV lamp while the vanillin-sulfuric acid reagent and heating gun were used for further visualisation. High-performance liquid chromatography (HPLC) was used for further purification process by using the JAI Recycling HPLC with model no. of LC-9130 NEXT. It was a semi preparative HPLC, smart and compact instrument since all-in-one body. This instrument has panel to control the injector, detector, pump and fraction collector from the computer or LCD touch screen panel. UV detector was used to detect four selected wavelengths from 200 nm to 800 nm. In this research, it favoured wavelength at 210 nm. Ten milligrams (10 mg) of the selected fractions with two or three spots on TLC were dissolved in 1 mL MeOH. Then, it was eluted with a mixture of MeOH: H_2_O with ratio of 60:40 in the presence of 0.1% formic acid as a buffer. Prior to analysis, all solvents were filtered with a nylon membrane filter that has a pore size of 0.45 μm. The fractions were eluted at a flow rate of 4.0 mL/min. Next, the NMR spectra were obtained by using a Bruker Advance 500 (500 MHz for ^1^H NMR, 125 MHz for ^13^C NMR) spectrometer system. Data were analysed via TopSpin 3.6.3 software package. Spectra were referenced to TMS or residual solvent (chloroform-d_1_ (CDCl_3_) = 7.26 ppm in ^1^H NMR and 77.2 ppm in ^13^C NMR; methanol-d_4_ (CD_3_OD) = 3.31 ppm, 4.87 ppm in ^1^H NMR and 49.3 ppm in ^13^C NMR). ^1^H NMR spectroscopic data is reported as follows: chemical shift (relative integral, multiplicity [s = singlet, d = doublet, dd = doublet of doublets, dt = doublet of triplets, t = triplet, m = multiplet] spin – spin coupling constant (*J* in Hertz, Hz). The HRESIMS were carried on a Thermoquest TLM Deca ion-trap mass spectrometer and Agilent 1290 Infinity Ultra-Performance Liquid Chromatography-Tandem Mass Spectrometer (UHPLC-MS/MS) by using direct infusion. Infrared (IR) spectra of the compounds were obtained using Perkin Elmer 2000 FT-IR at the School of Chemical Sciences, USM. All samples were analysed in the range of 4000 cm^−1^–600 cm^−1^ wavelengths to determine the functional group. The result formed a spectrum designated as% transmittance versus wavenumber (cm^−1^).

### Plant Material

The bark of *Beilschmiedia lumutensis* (*B. lumutensis*) was collected in Machang, Kelantan, Malaysia in July 2006. This species was identified by Teo L. E., a botanist from Department of Chemistry, Faculty of Science, University of Malaya. A voucher specimen (KL5269B) has been deposited at the Herbarium of the Department of Chemistry, Faculty of Science, University of Malaya, Kuala Lumpur, Malaysia for future reference.

### Extraction and Isolation of Compounds

The air-dried bark of *B. lumutensis* (1.5 kg) were sliced, ground into small pieces and extracted with EtOAc (3 × 1.5 L) followed by MeOH (3 × 1.5 L) at room temperature using maceration process. The solvents were evaporated under reduced pressure at 40°C to give 61.4 g (4.1%) of EtOAc and 69.2 g (4.4%) of MeOH extracts, respectively. The EtOAc crude extract (61.0 g) was subjected to CC (silica gel, 0.063 mm– 0.200 mm, *n*-hexane: EtOAc step gradient) to afford 10 fractions (BL_F1–F10). Selected fractions were subjected to semi-preparative recycling HPLC, using isocratic method with 60% MeOH:40% H_2_0 + 0.1% formic acid to give desired compounds. Fractions BL_F2 and BL_F3 were combined and further fractionated by using CC (silica gel, 0.040 mm–0.063 mm; *n*-hexane: EtOAc step gradient) to obtain 12 subfractions. BL_F2.1 (0.21 g) was then separated by using recycling HPLC (60% MeOH:40% H_2_0 + 0.1% formic acid) to afford lumutensic acid A (**1**, 1.8 mg). Next, BL_F2.3 (0.44 g) was further fractionated to give lumutensic acid B (**2**, 1.1 mg). BL_F2.5 weighed 0.58 g underwent further separation process by CC and yielded lumutensic acid C (**3**, 3.6 mg). The structure of isolated compounds were determined by spectroscopic analysis (See supporting information).

Lumutensic acid A (**1**): White solid; Yield: 1.8 mg (0.013%); FT-IR (ATR) V_max_ cm^−^^1^: 3421 cm^−^^1^ (hydroxyl group), 2922 cm^−^^1^ (C–H stretching symmetric CH_2_), 2854 cm^−^^1^ (C–H stretching asymmetric CH_2_) and 1705 cm^−^^1^ (carbonyl group); HRESIMS: *m/z* 316.1920 [M-H]^−^ (calc. mass 316.2402); ^1^H-NMR (500 MHz, MeOH-D4): See Table 1; ^13^C-NMR (125 MHz, MeOH-D_4_): See Table 1.

Lumutensic acid B (**2**): White solid; Yield: 1.1 mg (0.008%); HRESIMS: *m/z* 387.1438 [M+H]^+^ (calc. mass 387.3263); ^1^H-NMR (500 MHz, CDCI_3_): See Table 1; ^13^C-NMR (125 MHz CDCl_3_): See Table 1.

Lumutensic acid C (**3**): Yellowish; Yield: 3.6 mg (0.025%); HRESIMS: *m/z* 434.3249 (calc. mass 434.2457); ^1^H-NMR (500 MHz, CDCI_3_): See Table 1; ^13^C-NMR (125 MHz CDCl_3_): See Table 1.

### Molecular Docking

The molecular docking studies were performed according to the method described by the previous publications with some modifications (Mahdi *et al*. 2024a; 2024b; Mohamad *et al*. 2021; Radzuan *et al*. 2024; Saad *et al*. 2022b). Briefly, for the *in silico* assessment of anti-hyperglycemic, the receptors from the Protein Data Bank (PDB) database (https://www.rcsb.org/) were used, including the crystallographic structure of human pancreatic α-amylase in complex with nitrite and acarbose (PDB ID: 2QV4), which was resolved to a high resolution of 1.97 Å for α-amylase inhibition studies. For the α-glucosidase inhibition studies, the crystal of human intestinal C-terminal maltase-glucoamylase (ctMGAM) in complex with acarbose (PDB ID: 3TOP) was used with a resolution of 2.88 Å for molecular docking analysis. These two protein receptors were prepared using the DockPrep tools in UCSF Chimera (Regents of University of California, CA, USA) graphical user interface (GUI) (Pettersen *et al*. 2004) by removing water molecules, unrelated heteroatoms and co-crystal complexes. Protein preparation continued with the addition of polar hydrogens, the merger of non-polar hydrogens, and the addition of solvation parameters and Gasteiger charges, which were saved in .pdbqt format. In addition, the 3D structure of the compounds was sketched using ChemDraw Professional 23.0 (Revvity Signals Software, MA, USA), followed by ligand preparation and energy minimisation using the MM2 force field in the Chem3D tools, which were then saved as .pdb. The grid boxes were placed around co-crystallised ligand, which was acarbose for both α-amylase α-glucosidase. Specifically, docking for α-amylase was conducted using a grid box parameter centred at coordinates 14.5, 48.5 and 29.0 along the X, Y and Z axes, respectively, with a spacing of 0.375 Å and a grid size of 25 × 25 × 25 Å (X, Y and Z) to ensure comprehensive coverage of the active site of the enzyme. For α-glucosidase, the centre of the grid box was set to −30.6, 35.6 and 26.5 along the X, Y and Z axes, with a spacing of 0.375 Å and a grid box size of 30 × 30 × 30 Å (X, Y and Z). Computational docking simulations were carried out using the binding analysis/docking module of UCSF Chimera-AutoDock Vina (Eberhardt *et al*. 2021; Trott & Olson 2010). Ligand-receptor docking results from AutoDock Vina were further analysed and visualised in 2D and 3D conformations through BIOVIA Discovery Studio Visualizer 2024 Client (Dassault Systems, CA, USA).

## RESULTS AND DISCUSSSION

**FIGURE 2:**
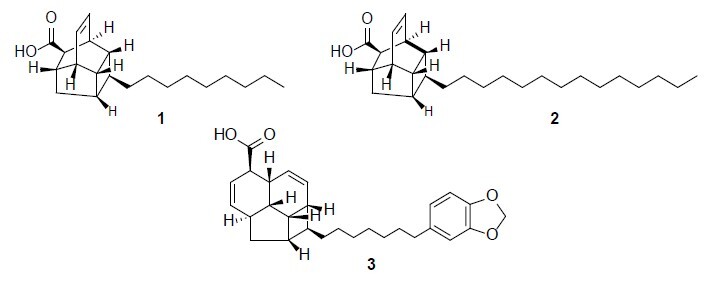
The structures of endiandric acids **1**–**3** isolated from the bark of *B. lumutensis*.

Phytochemical studies identified three compounds classified as endiandric acid derivatives: two endiandric acid type A compounds, lumutensic acid A (**1**) and lumutensic acid B (**2**), and one endiandric acid type B compound, lumutensic acid C (**3**), as shown in Fig. 2. All spectroscopic data are discussed in this section, and the NMR spectra are provided in the Supplementary Materials.

**FIGURE 3:**
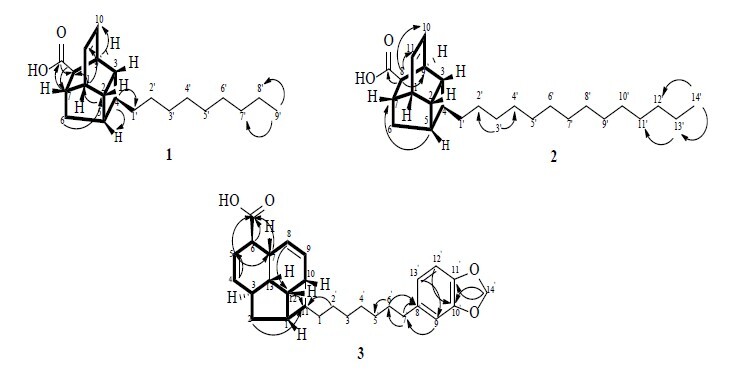
^1^H-^1^H COSY (bold) and HMBC (^1^H→^13^C) correlations of endiandric acids **1**–**3**.

Compound **1** was obtained as a white solid. It was classified as endiandric acid type A with molecular formula of C_21_H_32_O_2_, assignable on the basis of HRMS data by a molecular ion peak at [M+H]^+^ at m/z 316.1920 (calcd. for 316.2402) with 6 degrees of unsaturation. The IR showed absorption band at λ_max_; 3421 cm^−1^, 2922 cm^−1^, 2854 cm^−1^ and 1705 cm^−1^ indicating the presence of hydroxyl group, C–H stretching (sp^3^ hybridisation) for symmetric CH_2_, C–H stretching (sp^3^ hybridisation) for asymmetric CH_2_ and carbonyl group, respectively. Based on ^1^H NMR spectrum (Table 1), the unique characteristics of endiandric acid type A revealed at δ_H_ 6.14 (*J* = 7.1 Hz) and δ_H_ 6.24 (*J* = 7.3 Hz). Both peaks appeared as triplet which representing the *cis* double bond at H-10 and H-11. Next, a triplet signal at δ_H_ 0.91 (*J* = 6.6 Hz) confirmed the presence of terminal methyl. An alkyl chain can be affirmed by a broad peak resonated at δ_H_ 1.32. In addition, the ^13^C NMR and DEPT135 spectra showed 21 signals: 10 methines, nine methylenes, one methyl and one quaternary carbon (Table 1). The core structure which known as ‘cage like structure’ were represented by 11 skeletal signals resonated at δ_C_ 43.5 (C-1), 41.6 (C-2), 40.9 (C-3), 41.2 (C-4), 41.9 (C-5), 39.9 (C-6), 40.6 (C-7), 52.0 (C-8), 36.9 (C-9), 131.5 (C-10) and 133.9 (C-11). Eight methylenes carbon resonated between δ_C_ 23.82 to 33.17 confirmed the alkyl chain. Since the proton signals of alkyl group was broad, the positions of C1′ to C8′ were determined by the shielding and deshielding effects in carbon spectrum. C1′ was more deshielded than C2′ due to its position which was more adjacent to the electron withdrawing group. The further the alkyl carbon from the functional group, the lower the chemical shift. The linkage between the core structure was validated by the ^1^H-^1^H COSY spectrum (Fig. 3). The correlations between H-1/H-2, H-2/H-5, H-5/H-6β, H-6β/H-7, H-7/H-1 and H-8/H-9, H-9/H-3, H-3/H-2, H-2/H-1, H-1/H-7 and H-7/H-8 portrayed the existence of a cyclopentane (C1-C2-C5-C6-C7) and cyclohexane (C9-C3-C2-C1-C7-C8). In addition, a cyclobutene (C2-C3-C4-C5) and a six membered ring (C10-C11-C1-C2-C3-C9) were demonstrated by the correlations of H-10/H-11, H-11/H-1, H-1/H-2, H-2/H-3, H-3/H-9, H-9/H-10 and H-2/H-3, H-3/H-4, H-4/H-5 and H-5/H-2, respectively. The existence of carboxylic acid group was affirmed by the cross peak showed in HMBC. The correlations between H-7/C=O, H-8/C=O indicate that the position of carboxylic was bonded to C-8. Compound **1** was deduced as (1*S*,2*R*,3*R*,4*S*,5*S*,7*S*,8*R*,9*S*)-4-nonyltetracyclo[5.4.0.02,5.03,9]undec-10-ene-8-carboxylic acid also known as lumutensic acid A and it was a new endiandric acid type A.

Compound **2** was obtained as white solid. It was classified as endiandric acid type A. The HRMS spectrum of compound **2** revealed a molecular ion peak in a positive mode [M+H]^+^ at m/z 387.1438 (calc. mass 387.3263) corresponding with molecular formula of C_26_H_42_O_2_ and 6 degrees of unsaturation. The ^1^H NMR spectrum of compound **2** revealed the key features of ‘cage like structure’ with overlapping two triplets signal at δ_H_ 6.23, corresponding to *cis* olefinic protons at C-10 and C-11 (Table 1). Similar to compound **1**, terminal methyl was confirmed by the presence of triplet peak resonated at δ_H_ 0.93. A broad peak at δ_H_ 1.25 proved the occurrence of a long alkyl chain representing nine protons at H-4′-H-12′. Based on ^13^C and DEPT135 spectra, there were 26 signals with 10 methines, 14 methylenes, one methyl and one quaternary carbon (Table 1). The carboxylic acid was presented by quaternary signal resonated at δ_C_ 177.5. Ten methines resonated at δ_C_ 42.1 (C-1), 40.3 (C-2), 39.7 (C-3), 39.8 (C-4), 40.5 (C-5), 38.6 (C-7), 48.9 (C-8), 35.3 (C-9), 131.6 (C-10), 132.1 (C-11) and a methylene resonated at δ_C_ 38.7 (C-6). A long alkyl chain for compound **2** were confirmed by carbon signal in the range of δ_C_ 14.3 to 36.5. Carbon at C-1′ had higher chemical shift than neighbouring C-2′, C-3′ and so forth because of the deshielding effects. The ^1^H-^1^H COSY and HMBC correlation helped to determine the positions of the carbon and proton in this compound (Fig. 3). The cross peaks between H-8/C=O, H-7/C=O, H-8/C-O approved the position of carboxylic acid bonded to C-8. The neighbouring protons in the main skeleton were confirmed by the corelations of H-9/H-10, H-11/H-1, H-9/H-8, H-9/H-3, H-7/H-6, H-2/H-3, H-6/H-5, H-5/H-4, H-7/H-1, H-2/H-1 and H-9/H-3. Finally, with the aid from 1D and 2D spectroscopic data and published data, compound **2** was confirmed to be (1*S*,2*R*,3*R*,4*S*,5*S*,7*S*,8*R*,9*S*)-4-tetradecyltetracyclo[5.4.0.02,5.03,9]undec-10-ene-8-carboxylic acid known as lumutensic acid B and it was also a new endiandric acid type A.

Compound **3** was obtained as yellowish oil. It was classified as endiandric acid type B with molecular formula of C_28_H_34_O_4_ was determined by HRMS analysis in a positive mode [M+H]^+^ with molecular ion peak at m/z 434.3249 (calc. mass 434.2457), consistent with 12 degrees of unsaturation. The ^1^H NMR spectrum revealed the unique characteristics features of endiandric acid type B with *cis* double bonds at δ_H_ 6.22 (dt, *J* = 9.7, 2.5 Hz), 5.74 (dt, *J* = 9.7, 2.9 Hz), 5.46 (dt, *J* = 3.7, 10.1 Hz) and 5.64 (br dt, *J* = 3.7, 10.1 Hz) assignable to C-4, C-5, C-8 and C-9 (Table 1). Next, the presence of a benzene ring can be assured by three proton signals resonated at δ_H_ 6.66 (d, *J* = 1.0 Hz) 6.71 (d, *J* = 7.8 Hz) and 6.61 (dd, *J* = 7.8, 1.0 Hz) representing C-9′, C-12′ and C-13′, respectively. A singlet peak resonated at δ_H_ 5.91 portrayed the presence of methylenedioxyphenyl group. Meanwhile, the ^13^C NMR and DEPT135 spectra showed the presence of 16 methines and nine methylenes (Table 1). Four *cis* form alkene carbon signals resonated at δ_C_ 134.7 (C-4), 124.1 (C-5), 129.7 (C-8) and 129.8 (C-9) confirmed the key features for endiandric acid type B. One out of four quaternary carbon revealed by ^13^C NMR illustrated the presence of carboxylic acid, COOH group which resonated at δ_C_ 179.3. Three methine carbon resonated at δ_C_ 109.0 (C-9′), 108.20 (C-12′) and 121.20 (C-13′) represents the aromatic ring while one methylene carbon resonated at δ_C_ 100.9 showed the presence of methylenedioxyphenyl substituent. The 2D NMR spectra (HSQC, HMBC and COSY) were further studied to confirm the structure (Fig. 3). The HMBC correlations revealed the position of COOH group at C-6 through the cross peaks, of H6/C=O, H-5/C=O and H-7/C=O. Besides, the methylenedioxyphenyl moiety attached to C-8′ was confirmed by the HMBC correlation of H-13′/C-7′, H-9′/C-7′, H-6′/C-8, H-7′/C-6′and H-7′/C-8′. COSY correlation as shown in Fig. 3 with correlations at H-12/H-1, H-3/H-1, H-5/H-6, H-9/H-7, H-4/H-5, H-4/H-6 and H-4/H-3. On a final note, on comparison with spectroscopic data and literature, compound **2** was elucidated as (1*S*, 2*S*, 3*R*, 6*R*, 7*R*, 10*S*, 11*S*, 12*S*)-2-(1,3-benzodioxol-5-ylheptyl)tetracyclo[8.2.1.03,12.06,11]trideca-4,8-diene-7-carboxylic acid also known as lumutensic acid C and this is a new endiandric acid type B found in *B. lumutensis*.

The EtOAc extract was evaluated to α-amylase and α-glucosidase assays and it shows a good inhibition with IC_50_ values 7.90 ± 1.00 μg/mL and 21.91 ± 3.93 μg/mL, respectively (Tay *et al*. 2020). Due to limited amount of compounds, we decided to perform a molecular docking study to understand the free binding energy and interaction modes between the residues in the active site of the enzymes and compounds **1**–**3**.

Molecular docking simulations were conducted to calculate the theoretical binding energy and to simulate the binding interactions of the isolated compounds **1**–**3** in the hyperglycemic enzymes of α-amylase (PDB ID: 2QV4) and α-glucosidase (PDB ID: 3TOP). α-Amylase is a key enzyme in the breakdown of carbohydrates into glucose. By inhibiting this enzyme, the rate of glucose uptake can be reduced, which has a positive effect on the control of blood sugar levels in diabetics (Maurus *et al*. 2008). Besides, the MGAM protein is a type of α-glucosidase enzyme that is crucial in the final steps of carbohydrate digestion by breaking down starch into glucose. The C-terminal MGAM (ctMGAM) was selected as the docking protein for α-glucosidase as it has been reported to be favoured over the N-terminal when inhibited with acarbose (Phongphane *et al*. 2023). Of note, inhibition of this enzyme may help to control postprandial blood glucose levels, which is essential for the control of diabetes (Ren *et al*. 2011).

**FIGURE 4:**
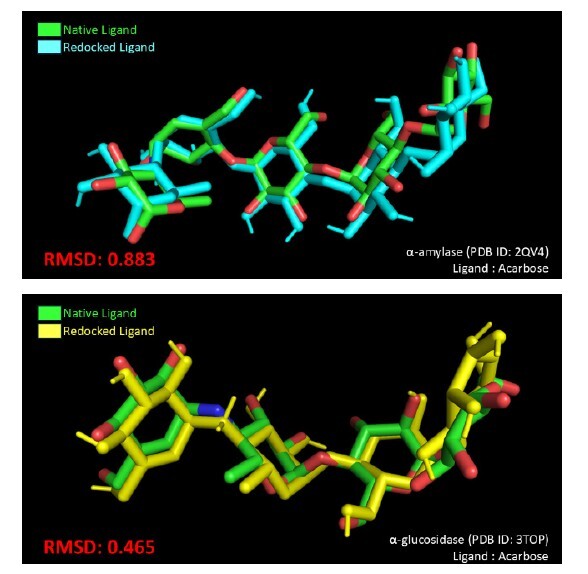
Root mean square deviation (RMSD) for α-amylase (top) and α-glucosidase (bottom).

Before initiating the molecular docking simulation, the docking parameters were validated by redocking the co-crystallised (native) ligands, namely acarbose, to the active site of these enzymes. The calculated root mean square deviation (RMSD) between the native and redocked ligands (Fig. 4) yielded values of 0.883 Å (α-amylase) and 0.465 Å (α-glucosidase), which are well below the acceptable threshold of 2.00 Å, indicating that the docking procedure is valid and can be used for compound docking (Bell & Zhang 2019; Zheng *et al*. 2022). Additionally, even with its bulky planar structure, acarbose forms stable complexes by directly interacting with the catalytic amino acids in the active sites of α-amylase and α-glucosidase, causing their 3D structures to adopt guided conformations similar to synthetic molecules (Şahin *et al*. 2023). Hence, the binding energies and inhibition constant of α-amylase and α-glucosidase with the target compounds and the docking controls are listed in Table 2, while the type of the binding interactions is detailed in Tables 3 and 4. As shown in Table 2, the negative binding energy and low inhibition constant of all target compounds indicate that the interactions with the receptor of the enzymes are thermodynamically favourable with an appreciable binding energy (Basir *et al*. 2024).

**FIGURE 5:**
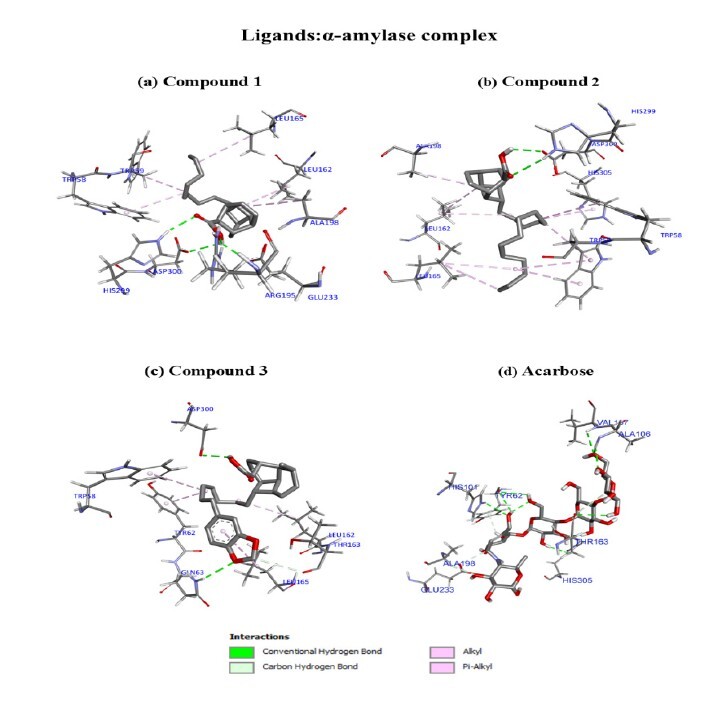
The three-dimensional binding modes of compounds **1**–**3** and acarbose are presented at the active site of α-amylase (2QV4).

In the docking evaluation of α-amylase, all isolated compounds exhibited a higher binding energy and lower binding affinity with the enzyme, ranging from −9.070 to −6.718 kcal/mol, compared to acarbose (docking control, standard drug) at −9.228 ± 0.010 kcal/mol. The molecular docking investigation of compound **1** revealed several interesting interactions with the binding site residues (Fig. 5a), with the ΔG of −6.964 ± 0.016 kcal/mol and K_i_ of 7.757 × 10^−6^. The residues of ARG195, GLU233 and ASP300 engaged in the conventional hydrogen bonds with the -OH moiety, while HIS299 formed a similar hydrogen bond with the carbonyl group of **1**, which stabilised the complex. In addition, two π-alkyl interactions were established between -CH_2_ moieties and the residues of TRP58 and TRP59. The cyclohexyl rings of **1** interact with LEU162 and ALA198 through alkyl interactions, whereas another -CH_2_ interacts with LEU165 through similar interactions.

In compound **2** (ΔG of −6.718 ± 0.074 kcal/mol; K_i_ of 1.175 × 10^−5^), the -C=O and -OH groups of this ligand formed conventional hydrogen bonds with HIS299 and ASP300, respectively, which contributes to the stabilisation of the ligand within the binding site of the enzyme (Fig. b). Moreover, the side chains of residues TRP58, TRP59 and HIS305 interact with the -CH_2_ hydrophobic regions of the ligand through π-alkyl interactions. Additionally, the alkyl side chain of LEU162, LEU165 and ALA198 interact with the alkyl groups of the ligand consisting of -CH_2_, -CH_3_ and the cyclohexyl ring through alkyl interactions, enhancing the hydrophobic interactions, which further contributed to the overall binding energy.

Compound **3** is the most stable docked complex with the highest binding affinity among the isolated compounds, with ΔG of −9.070 ± 0.128 kcal/mol and K_i_ of 2.209 × 10^−7^. It exhibited two conventional hydrogen bonds. The first is the oxygen of the methylenedioxyl with the amino group of GLN63 and the second is the -OH of the ligand with the ASP300 residue. Besides, the -CH_2_ of the methylenedioxyl participated in a carbon-hydrogen bond with the -C=O of ASP300, which drove the binding process. The π-alkyl interactions between TRP58, TYR62 and LEU165 with the alkyl segment of -CH_2_ and the phenyl moieties of the ligand further stabilised the complex, while an alkyl interaction occurred between -CH_2_ of **3** and LEU162 of the α-amylase enzyme (Fig. 5c).

**FIGURE 6:**
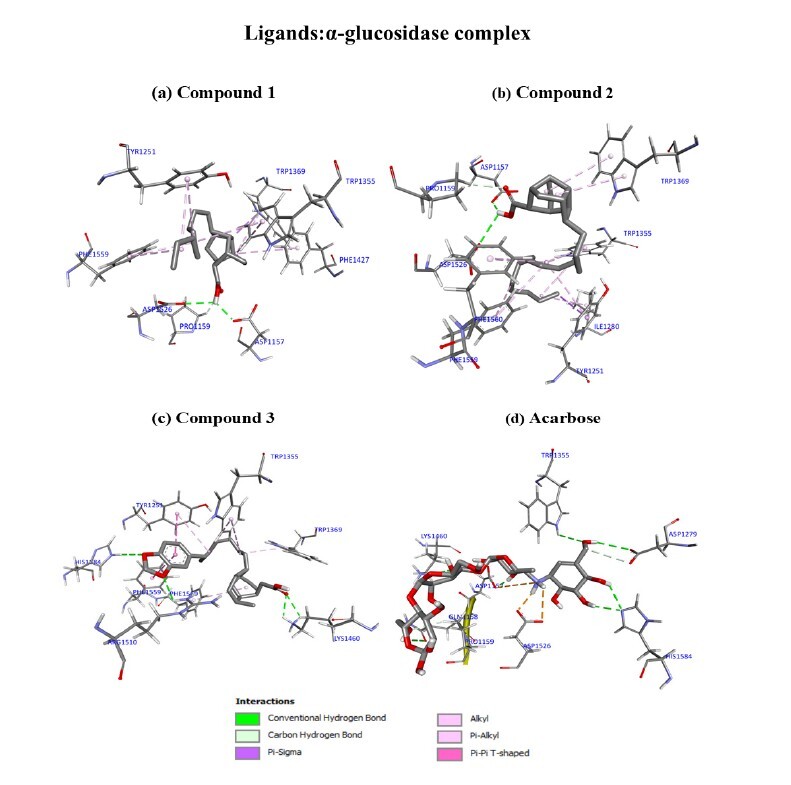
The three-dimensional binding modes of compounds **1–3** and acarbose are presented at the active site of α-glucosidase (3TOP).

Upon molecular docking of the α-glucosidase enzyme, all isolated compounds displayed comparable binding energy (in the range of −9.275 to −7.298 kcal/mol) as the acarbose control (−7.488 ± 0.129 kcal/mol). Of the three compounds analysed, compound **3** had the highest binding energy, followed by **1**. Compound **1** (ΔG of −7.553 ± 0.107; K_i_ of 2.867 × 10^−6^) unveiled several interactions with the residues of the α-glucosidase binding site, such as a conventional hydrogen bonding interaction between the -OH group of **1** and the carboxyl groups of ASP1157 and ASP1526, and a carbon-hydrogen bonding interaction between -C=O of **1** and the PRO1159 residue. Further, the π-system of the pyrrole ring in the indole group of TRP1369 and the cyclohexyl ring of **1** showed a π-sigma interaction, which increases the binding intercalation strength. Meanwhile, eight other hydrophobic π-alkyl interactions occurred between the cyclohexyl ring and the -CH_2_ moieties of the compound with TYR1251, TRP1355, TRP1369 and PHE1559, as shown in Fig. 6a.

In compound **2** (ΔG of −7.298 ± 0.062; K_i_ of 4.412 × 10^−6^), conventional hydrogen bonding were observed between the hydrogen atoms of the -OH group and the carboxyl of ASP1157 and ASP1526, while the -C=O in **2** interacts with the side chain of PRO1159 via a carbon hydrogen bond (Fig. 6b). Interestingly, a π-sigma interaction formed between the -CH_2_ group and the phenyl ring of the TYR1251 residue, which further stabilised the docked complex. In addition, the -CH_2_ units in **2** also formed another hydrophobic interaction via alkyl and π-alkyl with the enzyme residues of ILE1280, TYR1251, TRP1355, ASP1526, PHE1559 and PHE1560. Finally, two π-alkyl interactions occurred between the TRP1369 residue and the cyclopentyl and cyclohexyl ring structure of **2**. Remarkably, compound **3** is the most promising compound in the *in silico* α-glucosidase evaluation with a good binding energy of −9.275 ± 0.019 kcal/mol and K_i_ of 1.563 × 10^−7^ (Fig. 6c). This compound exhibited four conventional hydrogen bonds, the first two between the oxygen of -C=O with LYS1460 and two more between the oxygen of the methylenedioxyl group with ARG1510 and HIS1584. The next prominent interactions involved in this docked complex intercalation was π-π T-shaped between the phenyl group in **3** and the π-system of phenyl in residues TYR1251 and PHE1559, which contributed to the total binding energy. With the -CH_2_ group, four π-alkyl interactions were formed with TYR1251, TRP1355 and TRP1369. Another π-alkyl interaction was observed between the cyclohexyl ring of **3** with the protein residue PHE1560 on the phenyl ring.

## CONCLUSION

In this research, the phytochemical studies of ethyl acetate crude extracts of *B. lumutensis* yielded three new compounds: lumutensic acid A (**1**), lumutensic acid B (**2**) and lumutensic acid C (**3**). The molecular docking study revealed that all three isolated compounds exhibited potential inhibitory effects on the hyperglycemic enzymes α-amylase and α-glucosidase, with compound **3** showing the most promising results. Compound **3** had the highest binding affinity and stability for both enzymes, with strong hydrogen bonding and hydrophobic interactions contributing to its superior inhibitory potential. In the α-amylase docking, compound **3** demonstrated the most stable binding (ΔG of −9.070 kcal/mol), forming key interactions with active site residues like ASP300, TRP58 and LEU165. Similarly, for α-glucosidase, compound **3** showed the highest binding energy (ΔG of −9.275 kcal/mol), with multiple hydrogen bonds and hydrophobic interactions involving residues such as LYS1460, ARG1510 and HIS1584, outperforming the control drug acarbose. Compounds **1** and **2** also displayed favourable interactions but with lower binding energies, indicating reduced inhibitory potency. Overall, compound **3** emerges as a strong candidate for further development as a therapeutic agent for managing postprandial hyperglycemia in diabetes.

## Supplementary Information



## Figures and Tables

**TABLE 1:** ^1^H (500 MHz) and ^13^C (125 MHz) NMR data of compounds **1** (CD_3_OD), **2** and **3** (CDCl_3_).

Position	Compound 1		Compound 2		Compound 3	

	δ_H_ (*J* in Hz)	δ_C_	δ_H_ (*J* in Hz)	δ_C_	δ_H_ (*J* in Hz)	δ_C_
1	2.65 m	43.5	2.69 m	42.1	2.27 m	41.4
2	2.34 m	41.6	2.36 t (7.5)	40.3	1.33 dd (5.8, 12.8)	34.9
					1.58 m (5.3, 11.8)	
3	1.73 m	40.9	1.63 m	39.7	2.56 m	37.1
4	1.60 m	41.2	1.63 m	39.8	6.22 dt (9.7, 2.5)	134.7
5	2.23 m	41.9	2.22 br, d (6.3)	40.5	5.74 dt (9.7, 2.9)	124.1
6	1.58 m	39.9	1.55 d (12.7)	38.7	3.03 m	49.31
	1.91 m		1.90 m			
7	2.56 m	40.6	2.55 m	38.6	3.00 m	33.05
8	2.74 d (3.2)	52.0	2.88 d (3.6)	48.6	5.46 dt (3.7, 10.1)	129.7
9	2.99 m	36.9	3.03 m	35.3	5.64 br, dt (3.7, 10.1)	129.8
10	6.14 t (7.1)	131.5	6.23 m	131.6	2.26 m	35.1
11	6.24 t (7.3)	133.9	6.23 m	132.1	1.45 m	46.1
12	-	-	-	-	2.65 q (7.8)	33.2
13	-	-	-	-	1.72 m	42.2
1′	1.27 br	33.2	1.49 m	36.5	1.47 m	37.3
2′	1.32 m	30.9	2.35 m	33.7	1.22 m	27.1
3′	1.32 m	30.9	1.62 m	24.9	1.27 m	29.6
4′	1.32 m	30.8	1.25 m	29.9	1.27 m	29.3
5′	1.32 m	30.8	1.25 m	29.9	1.27 m	29.7
6′	1.32 m	30.5	1.25 m	29.8	1.56 m	31.9
7′	1.32 m	28.5	1.25 m	29.7	2.51 t (7.6)	35.9
8′	1.31 m	23.8	1.25 m	29.6	-	136.9
9′	0.91 t (6.6)	14.5	1.25 t (6.6)	29.5	6.66 d (1.0)	109.0
10′	-	-	1.25 m	29.3	-	147.6
11′	-	-	1.25 m	27.5	-	145.5
12′	-	-	1.25 m	32.1	6.71 d (7.8)	108.2
13′	-	-	1.31 m	22.9	6.61 dd (7.8, 1.0)	121.2
14′	-	-	0.94 t (6.9)	14.3	5.91 s	100.9
C=O	-	181.7	-	179.5	-	179.4

**TABLE 2:** *In silico* binding energy of compounds **1**–**3** and acarbose on α-amylase (2QV4) and α-glucosidase (3TOP).

Protein	Compound	Binding energy, ΔG (kcal/mol)	Inhibition constant, K_i_ [K = exp(ΔG/RT)]
α-amylase (PDB ID: 2QV4)	**1**	−6.964 ± 0.016	7.757 × 10^−6^
	**2**	−6.718 ± 0.074	1.175 × 10^−5^
	**3**	−9.070 ± 0.128	2.209 × 10^−7^
	Acarbose (Docking control)	−9.228 ± 0.010	1.692 × 10^−7^
α-glucosidase (PDB ID: 3TOP)	**1**	−7.553 ± 0.107	2.867 × 10^−6^
	**2**	−7.298 ± 0.062	4.412 × 10^−6^
	**3**	−9.275 ± 0.019	1.563 × 10^−7^
	α-Acarbose (Docking control)	−7.488 ± 0.129	3.200 × 10^−6^

*Note*: Mean ± standard deviation for *n* = 3 experiments

**TABLE 3:** The key interactions of the compounds **1**–**3** and acarbose with the amino acids of α-amylase (2QV4).

Protein	Compound	Residue	Moiety	Types of interaction
α-amylase (PDB ID: 2QV4)	**1**	TRP58	-CH_2_	π-Alkyl
		TRP59	-CH_2_	π-Alkyl
		LEU162	Ring (Cyclohexyl)	Alkyl
		LEU165	-CH_2_	Alkyl
		ARG195	-OH	Conventional H-bond (1.99 Å)
		ALA198	Ring (Cyclohexyl)	Alkyl
		GLU233	-OH	Conventional H-bond (2.40 Å)
		HIS299	-C=O	Conventional H-bond (2.33 Å)
		ASP300	-OH	Conventional H-bond (2.59 Å)

	**2**	TRP58	-CH_2_	π-Alkyl
		TRP59	-CH_2_	π-Alkyl
			-CH_2_	π-Alkyl
		LEU162	-CH_2_	Alkyl
			Ring (Cyclohexyl)	Alkyl
		LEU165	-CH_2_	Alkyl
			-CH_3_	Alkyl
		ALA198	Ring (Cyclohexyl)	Alkyl
		HIS299	-C=O	Conventional H-bond (2.63 Å)
		ASP300	-OH	Conventional H-bond (2.53 Å)
		HIS305	-CH_2_	π-Alkyl

	**3**	TRP58	-CH_2_	π-Alkyl
		TYR62	-CH_2_	π-Alkyl
		GLN63	-O (Methylenedioxyl)	Conventional H-bond (2.63 Å)
		LEU162	-C_H_2	Alkyl
		THR163	-CH_2_ (Methylenedioxyl)	Carbon H-bond
		LEU165	Phenyl	π-Alkyl
		ASP300	-OH	Conventional H-bond (2.27 Å)

	Acarbose (Docking control)	TYR62	-OH	Conventional H-bond (2.86 Å)
			-OH	Conventional H-bond (2.75 Å)
			-OH	Carbon H-bond
		HIS101	-O	Conventional H-bond (2.69 Å)
		ALA106	-O	Conventional H-bond (2.14 Å)
			-O	Conventional H-bond (2.49 Å)
		VAL107	-O	Conventional H-bond (2.53 Å)
		THR163	-OH	Conventional H-bond (2.60 Å)
			-CH	Carbon H-bond
		ALA198	-O	Carbon H-bond
		GLU233	-CH	Carbon H-bond
		HIS305	-OH	Conventional H-bond (2.84 Å)

**TABLE 4:** The key interactions of the compounds **1**–**3** and acarbose with the amino acids of α-glucosidase (3TOP).

Protein	Compound	Residue	Moiety	Types of interaction
α-glucosidase (PDB ID: 3TOP)	**1**	ASP1157	-OH	Conventional H-bond (2.07 Å)
		PRO1159	-C=O	Carbon H-bond
		TYR1251	-CH_2_	π-Alkyl
			-CH_2_	π-Alkyl
		TRP1355	-CH_2_	π-Alkyl
			Ring (Cyclohexyl)	π-Alkyl
		TRP1369	Ring (Cyclopentyl)	π-Sigma
			Ring (Cyclohexyl)	π-Alkyl
		PHE1427	Ring (Cyclohexyl)	π-Alkyl
		ASP1526	-OH	Conventional H-bond (2.53 Å)
		PHE1559	-CH_2_	π-Alkyl
			-CH_2_	π-Alkyl

	**2**	ASP1157	-OH	Conventional H-bond (2.01 Å)
		PRO1159	-C=O	Carbon H-bond
		TYR1251	-CH_2_	π-Sigma
			-CH_2_	π-Alkyl
		ILE1280	-CH_2_	Alkyl
		TRP1355	-CH_2_	π-Alkyl
			-CH_2_	π-Alkyl
		TRP1369	Ring (Cyclopentyl)	π-Alkyl
			Ring (Cyclohexyl)	π-Alkyl
		ASP1526	-OH	Conventional H-bond (2.82 Å)
		PHE1559	-CH_2_	π-Alkyl
			-CH_2_	π-Alkyl
		PHE1560	-CH_2_	π-Alkyl

	**3**	TYR1251	Phenyl	π-π T-shaped
			-CH_2_	π-Alkyl
		TRP1355	-CH_2_	π-Alkyl
			-CH_2_	π-Alkyl
		TRP1369	-CH_2_	π-Alkyl
		LYS1460	-C=O	Conventional H-bond (2.36 Å)
			-C=O	Conventional H-bond (2.55 Å)
		ARG1510	-O (Methylenedioxyl)	Conventional H-bond (2.75 Å)
		PHE1559	Phenyl	π-π T-shaped
		PHE1560	Ring (Cyclohexyl)	π-Alkyl
		HIS1584	-O (Methylenedioxyl)	Conventional H-bond (2.79 Å)

	Acarbose (Docking control)	ASP1157	-NH_2_	Attractive charge
			-OH	Conventional H-bond (1.97 Å)
		GLN1158	-OH	Conventional H-bond (3.04 Å)
		PRO1159	-OH	Conventional H-bond (2.60 Å)
		ASP1279	-OH	Conventional H-bond (2.95 Å)
			-CH_2_	Carbon H-bond
		TRP1355	-OH	Conventional H-bond (3.05 Å)
		LYS1460	-O	Conventional H-bond (2.42 Å)
		ASP1562	-NH_2_	Attractive charge, salt bridge
			-NH_2_	Attractive charge, salt bridge
		HIS1584	-OH	Conventional H-bond (2.49 Å)
			-OH	Conventional H-bond (2.36 Å)
