# Supplementary material for: New Endiandric Acid from Beilschmiedia lumutensis and Their Molecular Docking Study as α-amylase and α-glucosidase Inhibitors
Source: Trop Life Sci Res. 2026 Mar 31;37(1):315–38. doi: 10.21315/tlsr2026.37.1.15 (PMC13127991; doi:10.21315/tlsr2026.37.1.15)
Supplement: Supplementary file 1 [file TLSR_37-1-315-supplementarymaterials.pdf]

TROPICAL LIFE SCIENCES RESEARCH

SUPPLEMENTARY MATERIALS

New Endiandric Acid from *Beilschmiedia lumutensis* and Their Molecular Docking Study as α-amylase and α-glucosidase inhibitors

[1]Nur Amirah Saad, [1]Muhammad Solehin Abd Ghani, [2]Mohammad Tasyriq Che Omar, [3]Mohd Azlan Nafiah, [4]Unang Supratman, [4]Desi Harneti, [5]Cécile Apel, [5]Marc Litaudon, [6]Azeana Zahari, [6]Khalijah Awang and [1]Mohamad Nurul Azmi*.

[1]Natural Products and Synthesis Organic Research Laboratory (NPSO), School of Chemical Sciences, Universiti Sains Malaysia, 11800 Minden, Penang, Malaysia.
[2]Biological Section, School of Distance Education, Universiti Sains Malaysia, 11800 Minden, Pulau Pinang, Malaysia
[3]Department of Chemistry, Faculty of Science and Mathematics, Sultan Azlan Shah Campus, Universiti Pendidikan Sultan Idris, Proton City 35950, Perak Darul Ridzuan, Malaysia
[4]Department of Chemistry, Faculty of Mathematics and Natural Sciences, Universitas Padjadjaran, 45363 Jatinangor, Indonesia.
[5]Institut de Chimie des Substances Naturelles, CNRS, UPR 2301, Université Paris-Saclay, 91198 Gif-sur-Yvette, France
[6]Department of Chemistry, Faculty of Science, University of Malaya, 50603 Kuala Lumpur, Malaysia

***Correspondence:** mnazmi@usm.my

© Penerbit Universiti Sains Malaysia

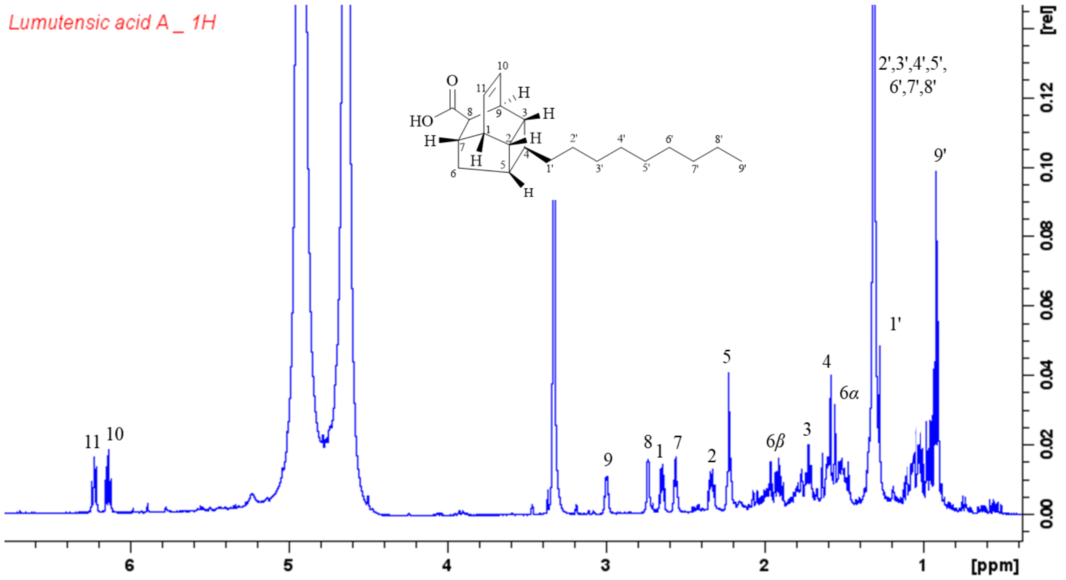

FIGURE S1: ^{1}H NMR spectrum (500 MHz, CD_3OD) for lumutensic acid A.

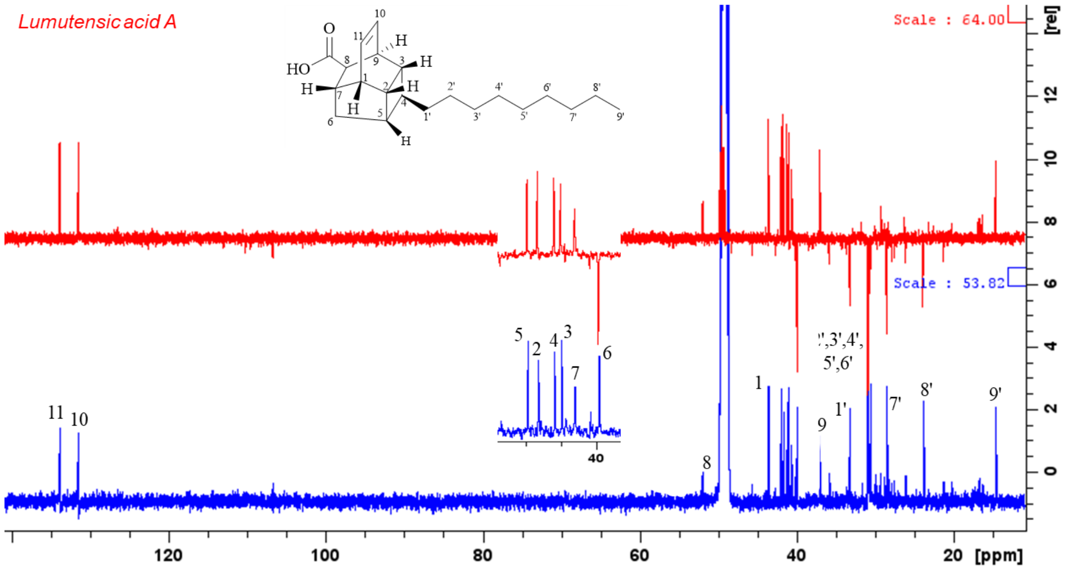

FIGURE S2: ^{13}C NMR (125 MHz, CD_3OD) and DEPT-135 spectrum for lumutensic acid A.

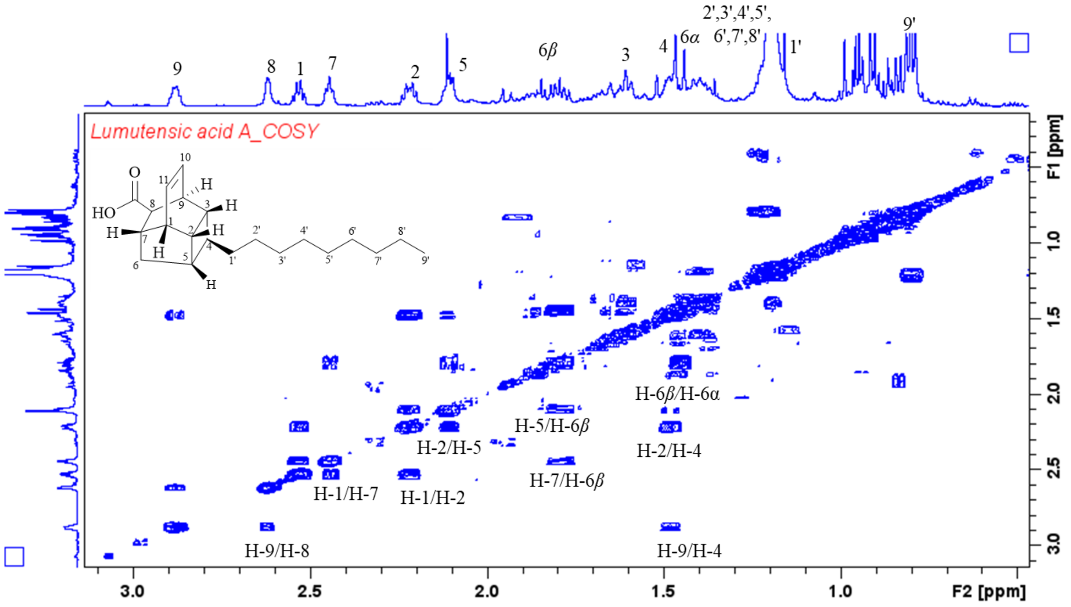

FIGURE S3: ^{1}H-^{1}H COSY spectrum for lumutensic acid A.

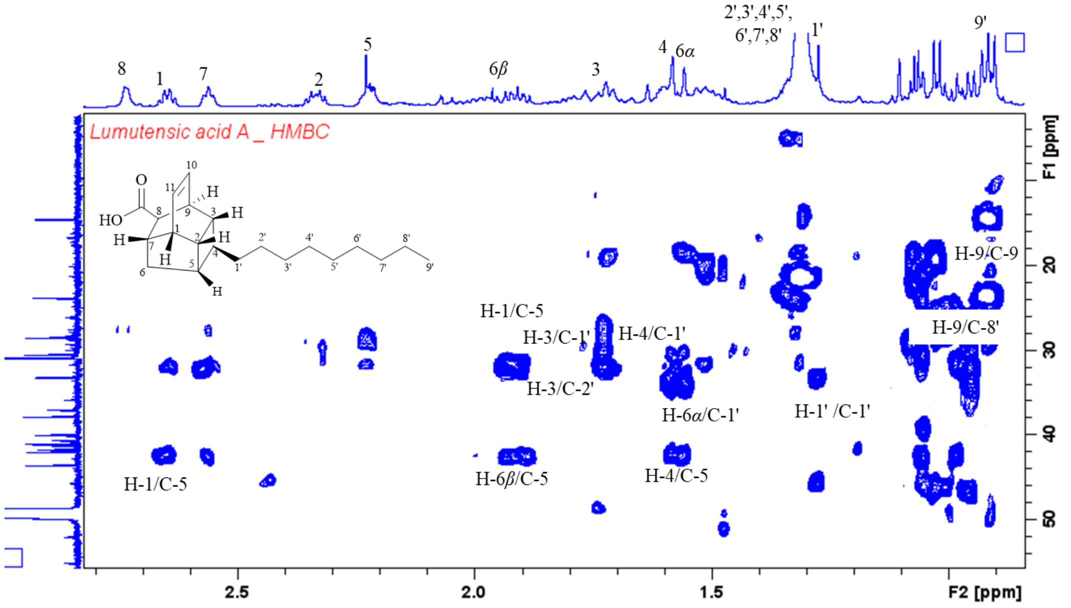

FIGURE S4: ^{1}H-^{13}C HMBC spectrum for lumutensic acid A.

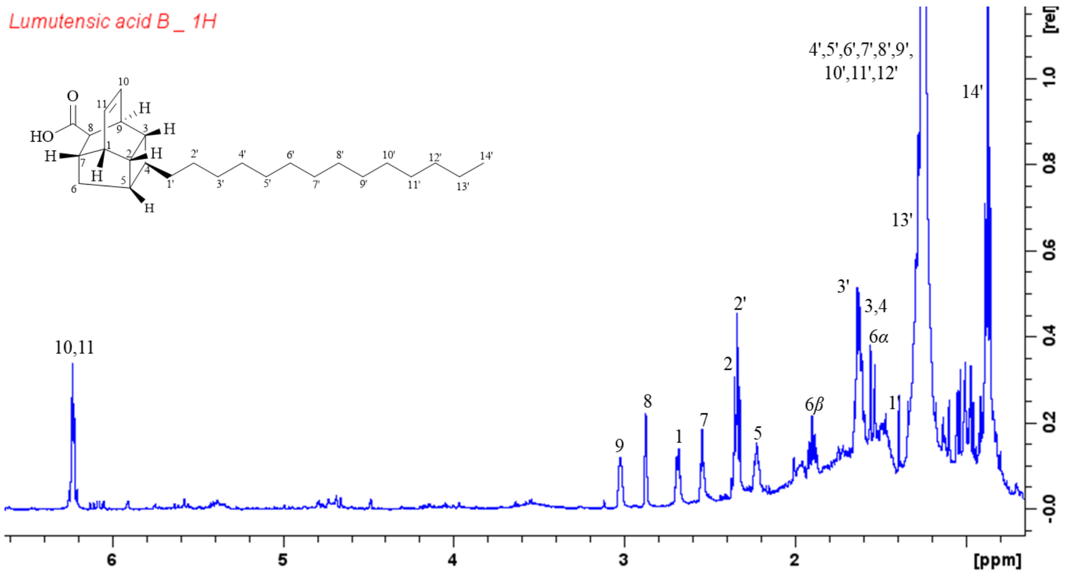

FIGURE S5: ^{1}H NMR spectrum (500 MHz, $CDCl_3$) for lumutensic acid B.

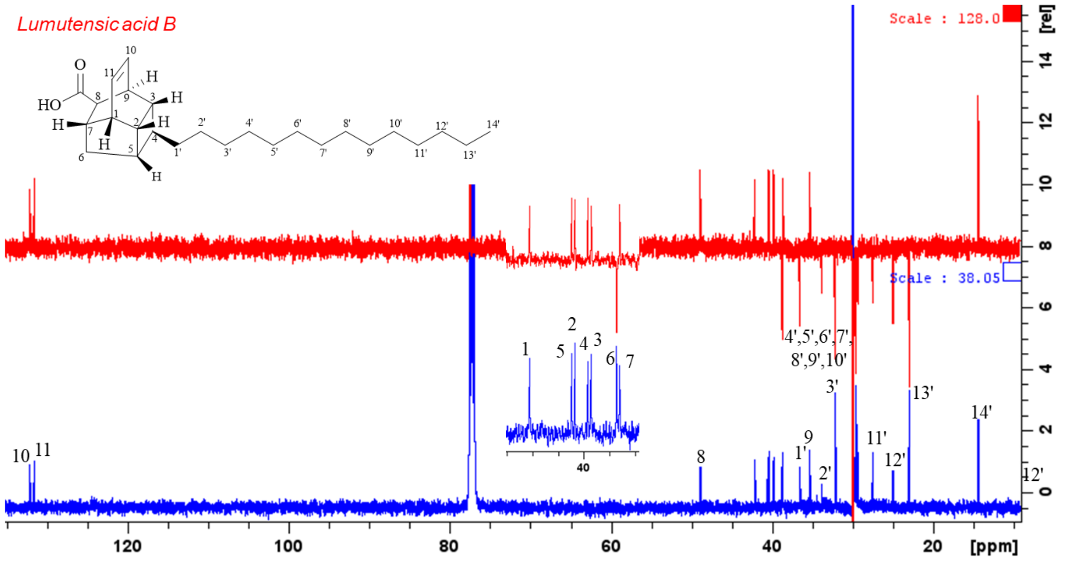

FIGURE S6: ^{13}C NMR (125 MHz, $CDCl_3$) and DEPT-135 spectrum for lumutensic acid B.

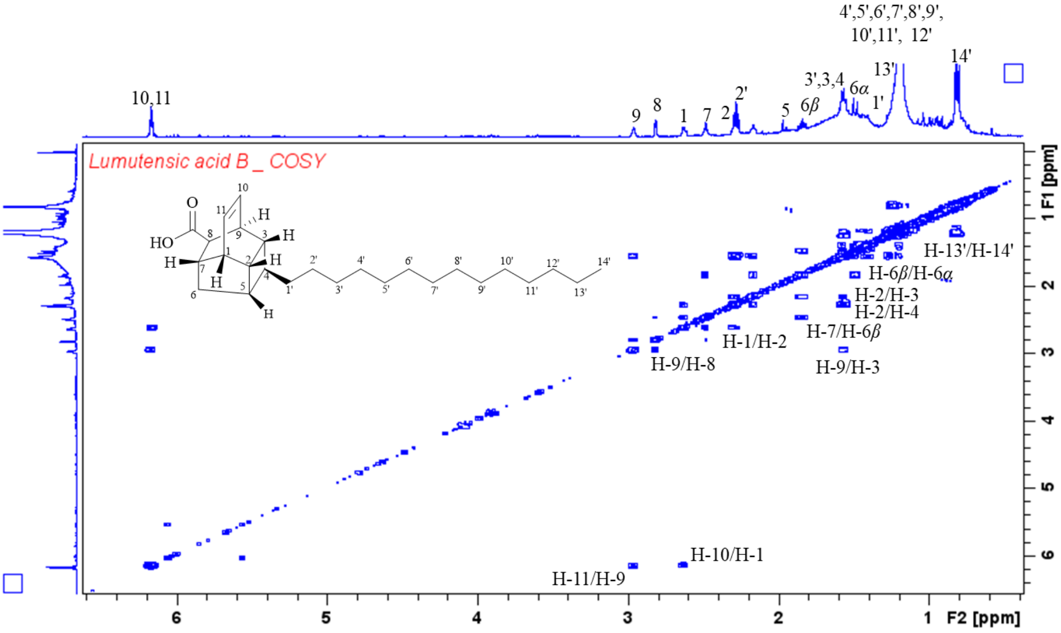

FIGURE S7: ^{1}H-^{1}H COSY spectrum for lumutensic acid B.

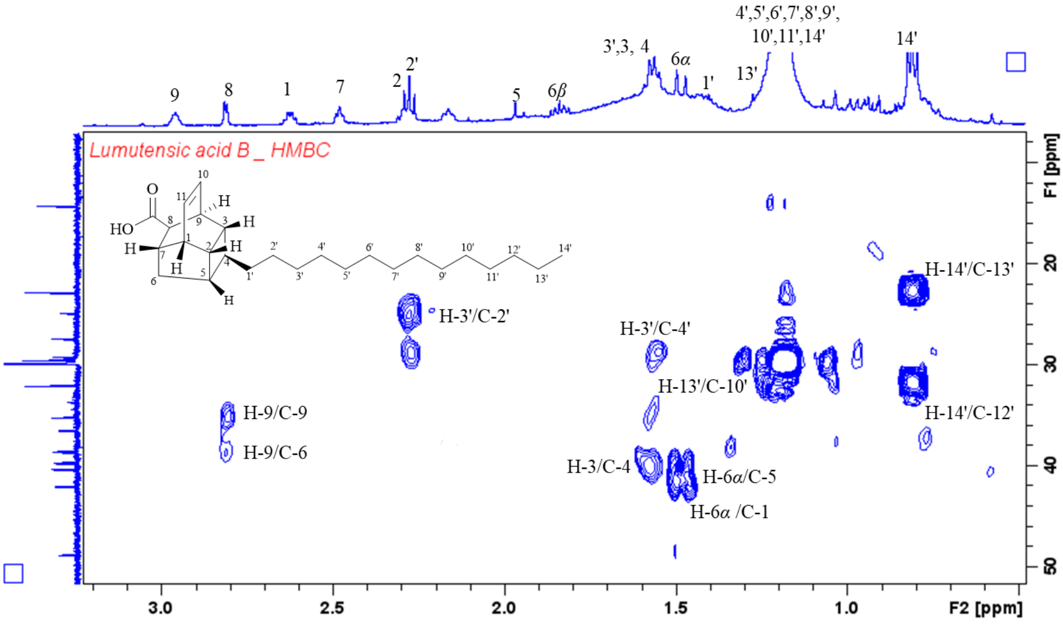

FIGURE S8: ^{1}H-^{13}C HMBC spectrum for lumutensic acid B.

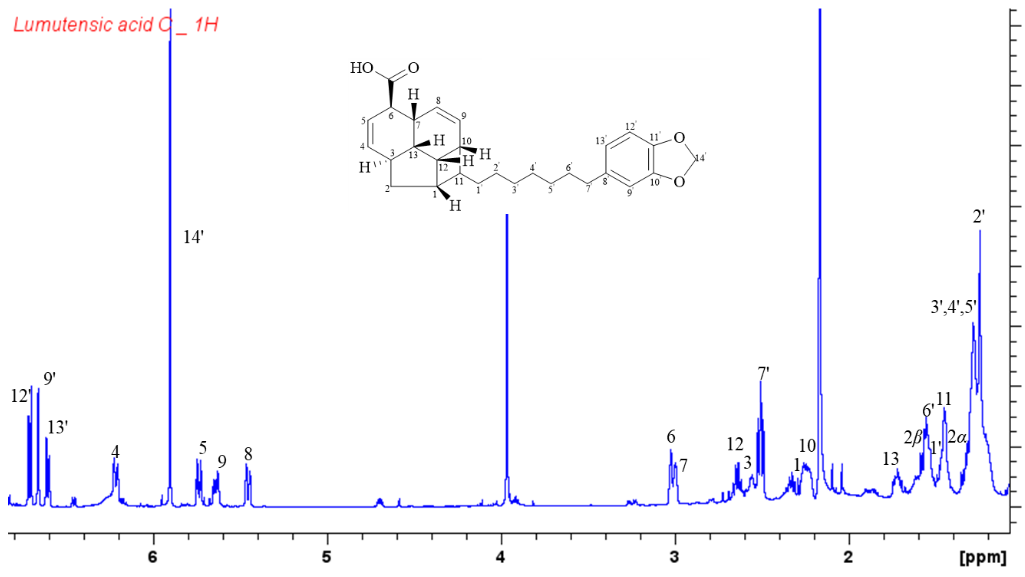

FIGURE S9: ^{1}H NMR spectrum (500 MHz, $CDCl_3$) for lumutensic acid C.

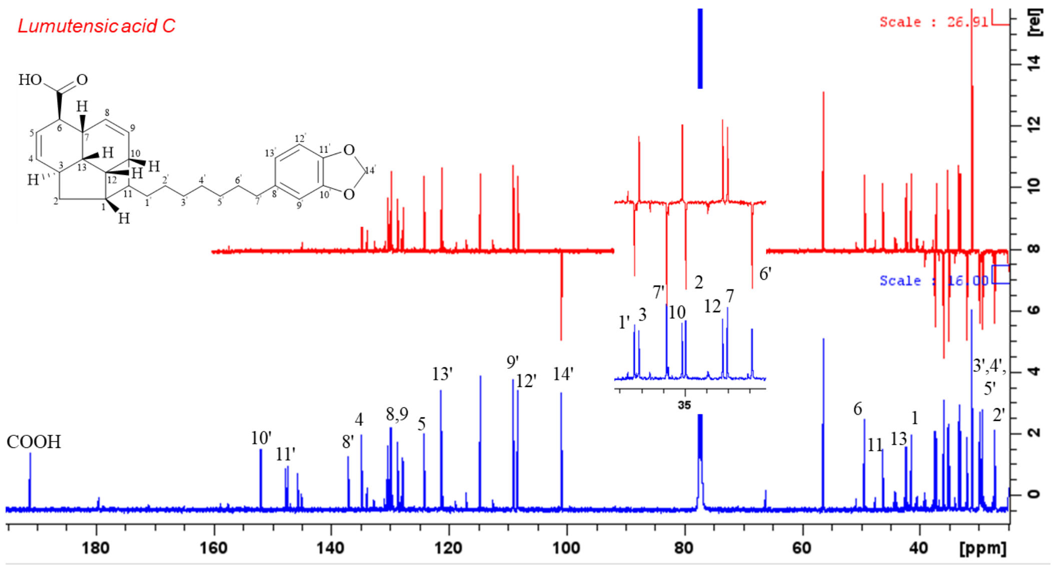

FIGURE S10: ^{13}C NMR (125 MHz, $CDCl_3$) and DEPT-135 spectrum for lumutensic acid C.

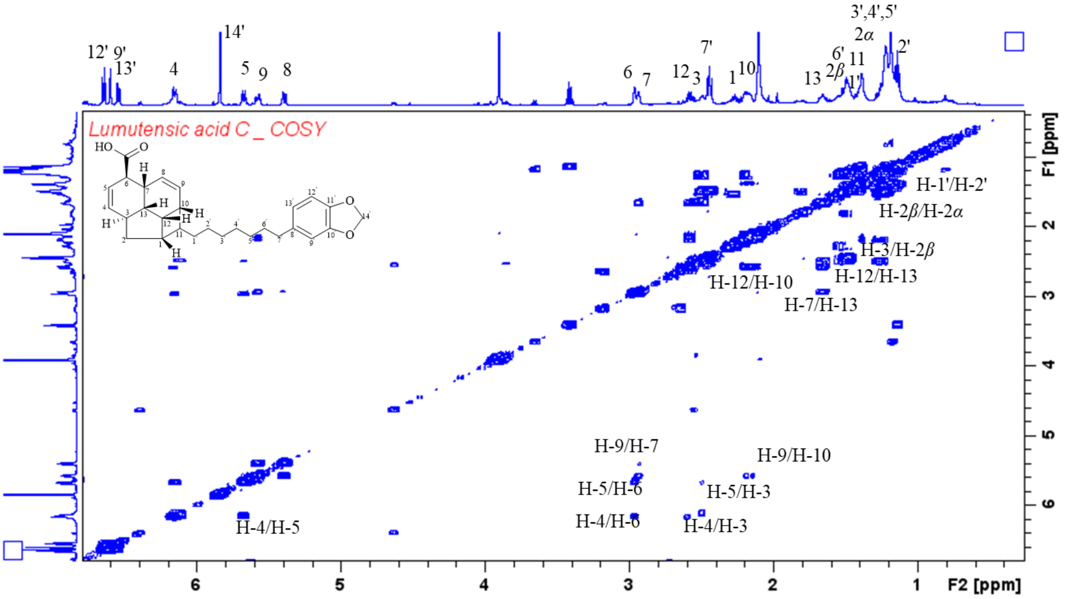

FIGURE S11: ^{1}H-^{1}H COSY spectrum for lumutensic acid C.

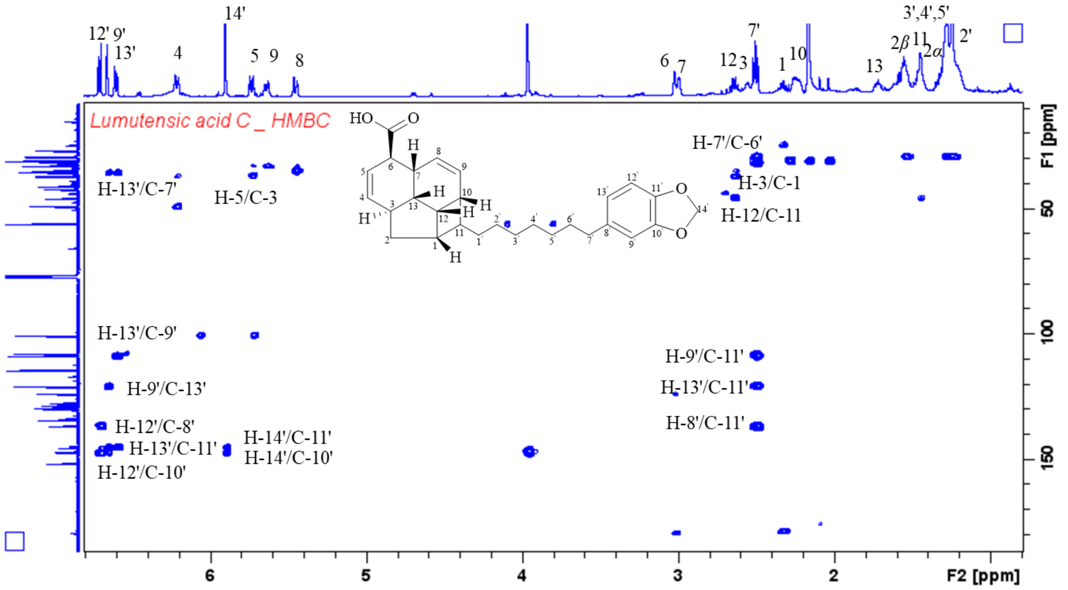

FIGURE S12: ^{1}H-^{13}C HMBC spectrum for lumutensic acid C.
